# Altered steering strategies for goal-directed locomotion in stroke

**DOI:** 10.1186/1743-0003-10-80

**Published:** 2013-07-22

**Authors:** Ala’ S Aburub, Anouk Lamontagne

**Affiliations:** 1 School of Physical and Occupational Therapy, McGill University, Montreal, Canada; 2 Jewish Rehabilitation Hospital Research site of the Center of Interdisciplinary Research in Rehabilitation of Greater Montreal (CRIR), Montreal, QC, Canada

**Keywords:** Heading, Hemiparesis, Optic flow, Virtual reality, Visuomotor, Walking

## Abstract

**Background:**

Individuals who have sustained a stroke can manifest altered locomotor steering behaviors when exposed to optic flows expanding from different locations. Whether these alterations persist in the presence of a visible goal and whether they can be explained by the presence of a perceptuo-motor disorder remain unknown. The purpose of this study was to compare stroke participants and healthy participants on their ability to control heading while exposed to changing optic flows and target locations.

**Methods:**

Ten participants with stroke (55.6 ± 9.3 yrs) and ten healthy controls (57.0 ± 11.5 yrs) participated in a mouse-driven steering task (perceptuo-motor task) while seated and in a walking steering task. In the seated steering task, participants were instructed to head or ‘walk’ toward a target in the virtual environment by using a mouse while wearing a helmet-mounted display (HMD). In the walking task, participants performed a similar steering task in the same virtual environment while walking overground at their comfortable speed. For both experiments, the target and/or the focus of expansion (FOE) of the optic flow shifted to the side (±20°) or remained centered. The main outcome measure was net heading errors (NHE). Secondary outcomes included mediolateral displacement, horizontal head orientation, and onsets of heading and head reorientation.

**Results:**

In the walking steering task, the presence of FOE shifts modulated the extent and timing of mediolateral displacement and head rotation changes, as well as NHE magnitudes. Participants overshot and undershot their net heading, respectively, in response to ipsilateral and contralateral FOE and target shifts. Stroke participants made larger NHEs, especially when the FOE was shifted towards the non-paretic side. In the seated steering task, similar NHEs were observed between stroke and healthy participants.

**Conclusions:**

The findings highlight the fine coordination between rotational and translational steering mechanisms in presence of targets and FOE shifts. The altered performance of stroke participants in walking but not in the seated steering task suggests that an altered perceptuo-motor processing of optic flow is not a main contributing factor and that other stroke-related sensorimotor deficits are involved.

## Introduction

Persons with stroke suffer from persistent mobility problems that are characterized by a slow walking speed [1], a lack of endurance [2] and a poor ability to adapt to the environment, such as when turning while walking (steering) to avoid an obstacle [3]. Approximately 40% of persons who suffered a stroke fall within the first year of a stroke [4,5]. Chances of having a hip fracture resulting from a fall during turning while walking would be eight times higher than a fall while walking forward [6]. These observations highlight the need to investigate in more details the nature of deficits leading to steering difficulties in persons with stroke.

In order to walk safely and efficiently in different or unfamiliar environments, one needs to know the spatial relationship between self and objects and be able to update this relationship as he/she moves. Vision provides rich information about self-motion and the characteristics of the environment. When walking towards a stationary goal, there are two main sources of information that are known to guide heading: the optic flow created by the self-motion of the participant [7] and the relative location of the goal (e.g. egomotion theory) [8]. Optic flow is defined as a radial pattern of light produced at the eye of the participant when moving through the environment [9]. It comprises of a point from which the motion radiates, known as the focus of expansion (FOE). According to the optic flow theory, a participant heads toward the desired direction by aligning the FOE with the goal or the target location [7].

The influence of optic flow on the control of locomotor heading has been extensively studied in healthy individuals [7,10,11]. Using a virtual reality set-up, studies demonstrated that when the FOE is artificially shifted to the side while walking, participants compensate by veering in the direction opposite to the shift in the physical world. This allows them to remain aligned with the desired goal location in the virtual world. In stroke participants, however, steering responses to changes in optic flow direction can be profoundly altered, leading to heading errors [12,13]. It was suggested that a defective visual motion perception, which can occur after stroke [14,15] could be a contributing factor. However, as steering control is normally investigated during locomotion, it remains difficult to differentiate the extent to which altered steering abilities result from the participants’ poor locomotor or sensorimotor function, and/or from an altered processing of visual motion information. Furthermore, previous locomotor paradigms that involved optic flow manipulations were devoid of visible target or goal [12,13], hence providing little information about goal-directed locomotion abilities in stroke survivors. As walking in an ecological environment most often involves steering towards stationary or moving goals, examining how individuals interact with changing optic flows and goal locations is needed.

The aim of this study was to compare stroke and healthy participants on their ability to control heading (steering) while exposed to changing optic flows and target locations. We previously developed a measure of heading performance (error) to capture how participants adapt their head/body horizontal orientation and location to align themselves with a target [16]. In this study, we examined and quantified the contribution of these variables to the locomotor steering performance displayed by the stroke and healthy participants. In order to better understand the impact of perceptuo-motor problems on steering performance, both groups of participants were also tested on a steering task performed in sitting with a computer mouse. This seated steering task, which was performed with the non-paretic hand by the stroke participants, allowed an evaluation of steering abilities while minimizing the impact of sensorimotor dysfunction and poor locomotor capacity due to stroke. It was hypothesized that stroke participants would display larger heading errors in the walking steering task compared to healthy controls, especially in presence of complex visual stimuli comprising of joint changes in optic flow and target locations. The larger heading errors would persist in the steering task performed while seated, suggesting a contribution of perceptuo-motor problems to the altered steering performance.

## Methods

### Participants

Ten participants with stroke and 10 healthy controls participated in this study (Table 1). All participants gave written informed consent and the project was approved by the Ethics Committee of CRIR. The inclusion criteria for the stroke participants were the following: age between 40–79 years, a first supratentorial stroke (≤ 12 months) in the middle cerebral artery territory, an ability to walk independently with or without a walking aid for at least 7 meters at a gait speed slower than 1 m/s, as well as the presence of a residual impairment in motor recovery for the leg and foot, as indicated by scores ≤6 out of 7 on the Chedoke-McMaster Stroke Assessment. Exclusion criteria included the presence of visual field defects (Goldman perimetry test) or visuospatial neglect (Star Cancellation Test and/or Line Bisection Test). Stroke and healthy participants were free of dementia or cognitive impairments (score ≥ 26 on the Mini-Mental Sate Examination), visual problems not corrected by eyewear and musculoskeletal or other neurological conditions interfering with locomotion.

**Table 1:** Participant characteristics

**Stroke**	**Age**	**Gender**	**Height**	**Weight**	Handedness*	Speed**	**MoCA†**	**Trail**	**Side**	**Chronicity**	**Chedoke**	**Chedoke**
**(n = 10)**	**(yrs)**	**(F/M)**	**(cm)**	**(Kg)**		**(m/s)**	**(/30)**	**Making B (s)**	**CVA**	**(mo)**	**Leg (/7)**	**Foot (/7)**
S1	58	M	176	68	R	0.28	NA	††	L	12	5	3
S2	43	M	168	70	R	0.6	24	120	R	12	3	3
S3	61	M	174	93	R	0.46	26	44	R	8	3	2
S4	45	M	183	84	R	0.55	29	66	L	6	4	3
S5	43	M	186	82	R	0.62	30	70	L	4	5	4
S6	58	F	170	72.7	R	0.26	23	118	L	7	3	2
S7	55	M	165	66	R	0.24	29	110	L	5	4	3
S8	57	M	183	84	R	0.85	30	68	L	7	5	4
S9	68	M	162	77.3	R	0.51	23	182	L	7	3	3
S10	68	F	176	76	L	0.82	30	87	L	3	5	5
**Mean**	**56**	**-**	**174**	**77**	**-**	**0.52**	**27**	**96**	**-**	**7**	**4**	**3**
**Range**	**43-68**	**8M/2F**	**162-186**	**68-93**	-	**0.28-0.82**	**23-30**	**44-182**	**-**	**3-12**	**3-5**	**2-5**
**Healthy**	**Age**	**Gender**	**Height**	**Weight**	**Handedness**	**Speed**	**MoCA**	**Trail**				
**(n = 10)**	**(yrs)**	**(F/M)**	**(cm)**	**(Kg)**		**(m/s)**	**(/30)**	**Making B (s)**				
**Mean**	**57**	**-**	**170**	**77**	-	**1.09**	**27**	**84**				
**Range**	**42-76**	**6M/4F**	**152-183**	**58-100**	**-**	**0.46-1.53**	**24-30**	**51-159**				

* Handedness assessed using the Edinburgh Handedness Inventory; ** Comfortable walking speed over 10 m; †Montreal Cognitive Assessment (MoCA); †† Trail Making B not evaluated for this first participant.

### Setup and procedure

Participants were tested on two steering tasks that were performed in walking (Experiment 1) and while seated (Experiment 2). Experiments took place in a random order in separate sessions, no more than one week apart.

### Experiment 1: Walking steering task

**Figure 1:**
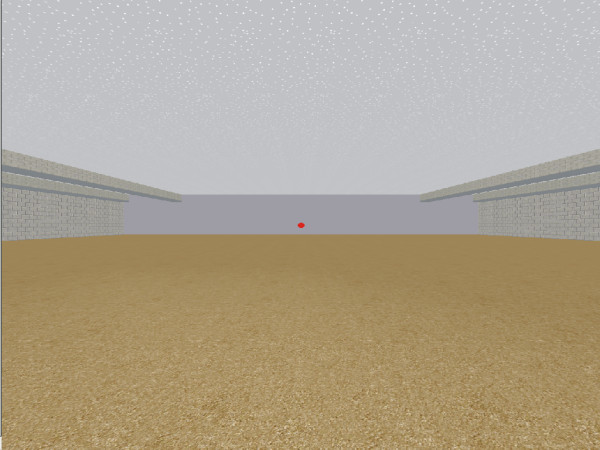
Screen shot of the virtual environment used for this experiment.

Participants were evaluated while walking overground in a large open space at their comfortable speed (stroke participants: 0.33 ± 0.21 m/s) or at slow speed (healthy participants: 0.57 ± 0.16 m/s). They were watching, in a Nvisor helmet mounted display (HMD), a virtual environment (VE) consisting of a large room (40 m × 25 m) with a target located in the center at eye level and 7 m away (Figure 1). The HMD had a 60˚ digonal field of view and 1.280 × 1.084 pixel resolution. Passive reflective markers were placed on specific body landmarks, as described in the Plug in Gait Model from Vicon. Head movement was represented by a 3-marker model, with markers located on the front, left side and right side of the HMD. Marker positions were recorded with a 12-camera motion capture system (Vicon) and fed to the CAREN-3 (Motek) virtual reality system. Head coordinates were used to update the subject’s perceived position, in real- time, in the virtual environment. The delay in updating the virtual environment in the HMD was less than 10 ms and the sampling frequency was set at 120 Hz.

When the participants achieved a forward walking displacement of 1.5 m, the target and FOE locations either remained at 0° or shifted ±20° to the right or left, in a random order. Participants were therefore exposed to a total of 9 experimental conditions that included 3 target locations and 3 optic flow locations. Participants were instructed to ‘walk toward the target’, hence covering a distance of 5 m after which the trial stopped. Five to 10 practice trials were provided depending on the needs and the endurance of the participants. In the healthy participants, 3 blocks of 9 trials were collected, for a total of 27 trials. Stroke participants performed 2 or 3 blocks depending on their endurance, except for one participant (S7) who performed only one block of 9 trials due to limited endurance. Rest was provided between each block and, when needed, within each block. Participants were accompanied by a physiotherapist while walking and no falls occurred during the experiment.

### Experiment 2: Seated steering task

Participants were assessed while seated and watching the same VE as in Experiment 1 in the HMD. The VE moved backward at 0.75 m/s, creating the illusion of walking forward. A speed of 0.75 m/s was chosen as a compromise between the comfortable gait speed displayed by stroke individuals and that of healthy age-matched controls (see Table 1). The same 9 experimental conditions as in the Experiment 1 were tested. Participants aligned themselves with the target in the VE using a computer optical mouse located on a table set at comfortable height (elbow 90°). The mouse was held either with the non-paretic (stroke) or dominant (healthy) hand and its degrees of freedom were restricted to the ML direction using a longitudinal, low friction physical stopper located on the table. Due to the side of CVA and handedness, 7 stroke participants performed the steering task with their non-dominant hand, and 3 with their dominant hand. After one complete block of practice trials, participants executed 6 blocks of 18 trials for a total of 108 trials. The purpose of this experiment was to rule out the contribution of sensorimotor impairments (e.g. weakness of paretic side, balance issues) which may affect the locomotor steering performance of stroke participants while providing insight into the possible role of an altered perception and integration of optic flow to guide steering.

### Data analysis

The primary outcome measure for the walking experiment was the net heading error (NHE) calculated in the virtual world’s coordinate. The NHE was calculated as the difference between the ideal and actual head in space orientation given the participants’ mediolateral (ML) position at 5 m of forward displacement. Since the extent of head rotation is dependent on the participants’ ML position, there are infinite possibilities of combination of centre of mass (CoM) ML displacement and head in space orientation to be perfectly aligned with the target and hence obtain a NHE of 0°. Secondary outcome measures included CoM ML displacement, head orientation, as well as the onset of head and heading reorientation in the virtual world’s coordinate. Heading was calculated as the participant’s real time virtual trajectory angle as they moved in the VE. Onset of heading reorientation was determined first by assessing the regression line of heading over time and the corresponding standard deviation (SD) before the perturbation started (0 m to 1.5 m). The regression line was then extrapolated beyond 1.5 m of forward displacement, and the onset of heading reorientation was identified as the first time point for which the difference between the real data and the regression line exceeded 3 SD, minus the time at which the perturbation was initiated (i.e. a 1.5 m of forward walking). A similar procedure was used to calculate the onset of head reorientation.

For the seated experiment, outcome measures included the NHEs, virtual ML displacement as well as onset of heading reorientation. NHEs were calculated as the angle between the participant’s virtual trajectory and the target, because participants only executed virtual ML displacements with the mouse. All variables other than onsets were recorded or calculated throughout the trials but the performance of participants on each of them is reported at 5 m of forward walking, that is at the end the trials. Data were analyzed for each trial before being averaged for each of the 9 conditions, and across participants. By convention, the paretic *side* of the stroke participants is represented as the *right* side (except when otherwise specified)*. Negative values* of ML displacement and head orientation are towards the *left,* whereas *positive* values took place towards the *right.*

### Statistical analysis

Absolute NHEs, the relative ML position of the participants with respect to the target, as well as onsets of head and heading reorientation were compared across conditions and groups using repeated measure ANOVAs with ‘target’ (3 locations) and ‘FOE’ locations (3 locations) as within-subject factors and ‘group’ as the between subject-factor (stroke vs. healthy). When statistically significant, ANOVAs were followed by post-hoc T-tests with Bonferroni adjustments. Head reorientation data were analyzed qualitatively due to the variety of behaviors that were observed, especially among the stroke participants. Pearson Correlation Coefficients were used to quantify the relationship between absolute NHEs during the walking tests and clinical measures of walking capacity (gait speed) and executive cognitive function (Trail Making B).

## Results

### Walking experiment

**Figure 2:**
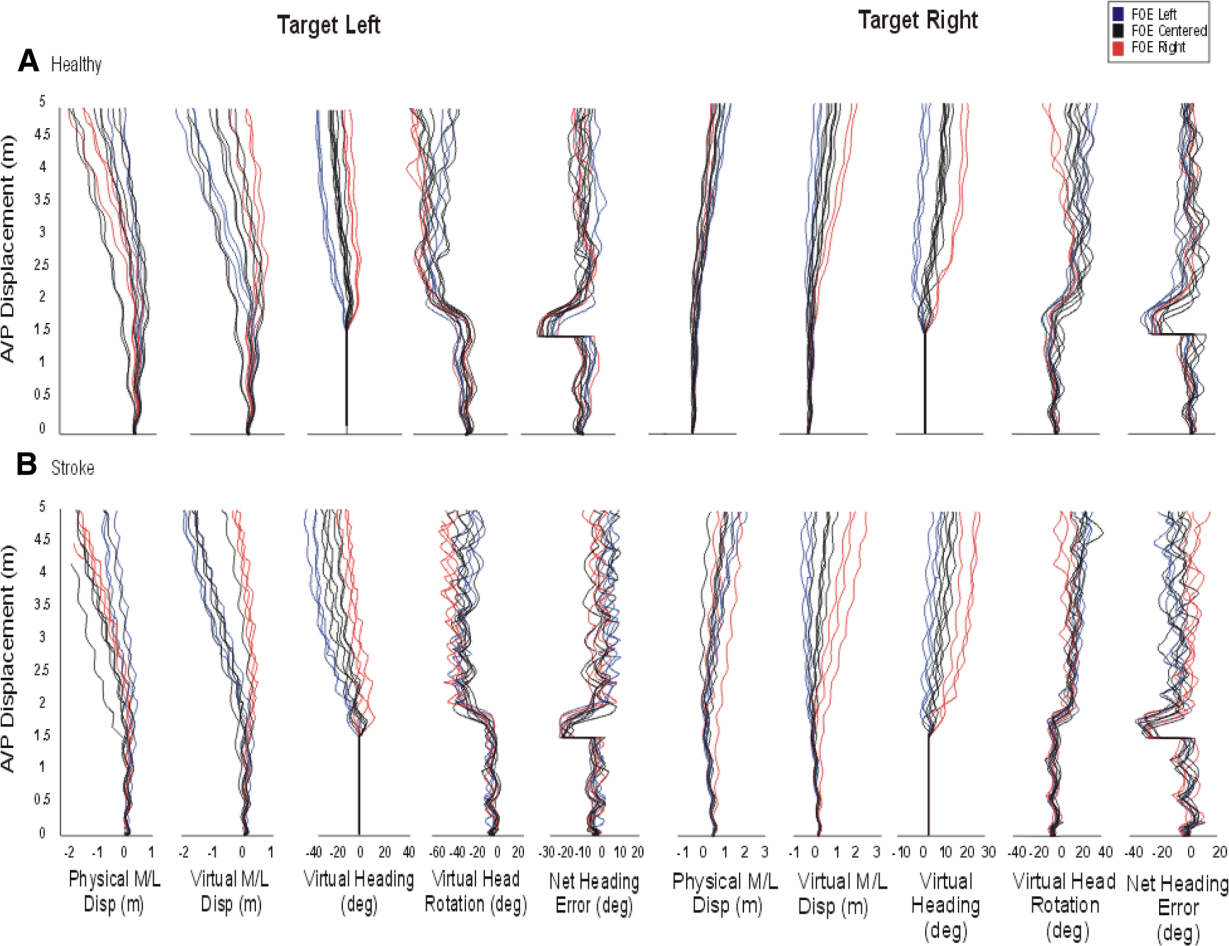
**Individual examples of walking steering behaviors for one healthy and one stroke participant exposed to target shifts towards the left and right under optic flows of different FOE locations.** A/p: anterio-posterior, M/L: Mediolateral, Disp: Displacement, Deg: Degree, FOE: focus of expansion.

Individual examples of steering behaviors for one healthy and one stroke participant exposed to target shifts towards the left and right under optic flows of different FOE locations (right, left, centre) are illustrated in Figure 2. When the target was shifted towards the left, the healthy participants’ trajectory veered towards the left, both in the physical and the virtual world coordinates. The direction of the flow, however, modulated the extent of this leftward deviation, such that a FOE on the right (contralateral to target location), which was creating the illusion of moving towards the right in the virtual world, accentuated the leftward ML trajectory corrections in the physical world. At variance, a FOE shifted to the left (ipsilateral to target location) resulted in smaller leftward ML displacement in the physical world. The amount of physical ML correction in presence of a flow shift, however, was not enough to ‘head’ directly on target. Indeed, while the participant’s virtual heading reached approximately 20° when there was no FOE shift (black traces), which corresponds to the target angular orientation, virtual heading magnitudes were smaller and larger, respectively, in presence of contralateral (red traces) and ipsilateral (blue traces) FOE shifts. Consequently, the participant executed larger head reorientations in presence of FOE shifts in order to be aligned with the target, which resulted in small NHEs across all conditions. The representative stroke participant displayed on Figure 2B shows adaptations in ML trajectory, heading and head orientation that were generally similar to healthy controls, at least in terms of the directions of these adaptations. This stroke participant, however, made smaller head reorientations which were not enough to be aligned with the target and resulted in larger NHEs across conditions compared to the healthy control participant, especially when the target was shifted to the right or paretic side.

### Medio-lateral displacement and head reorientation strategies

**Figure 3:**
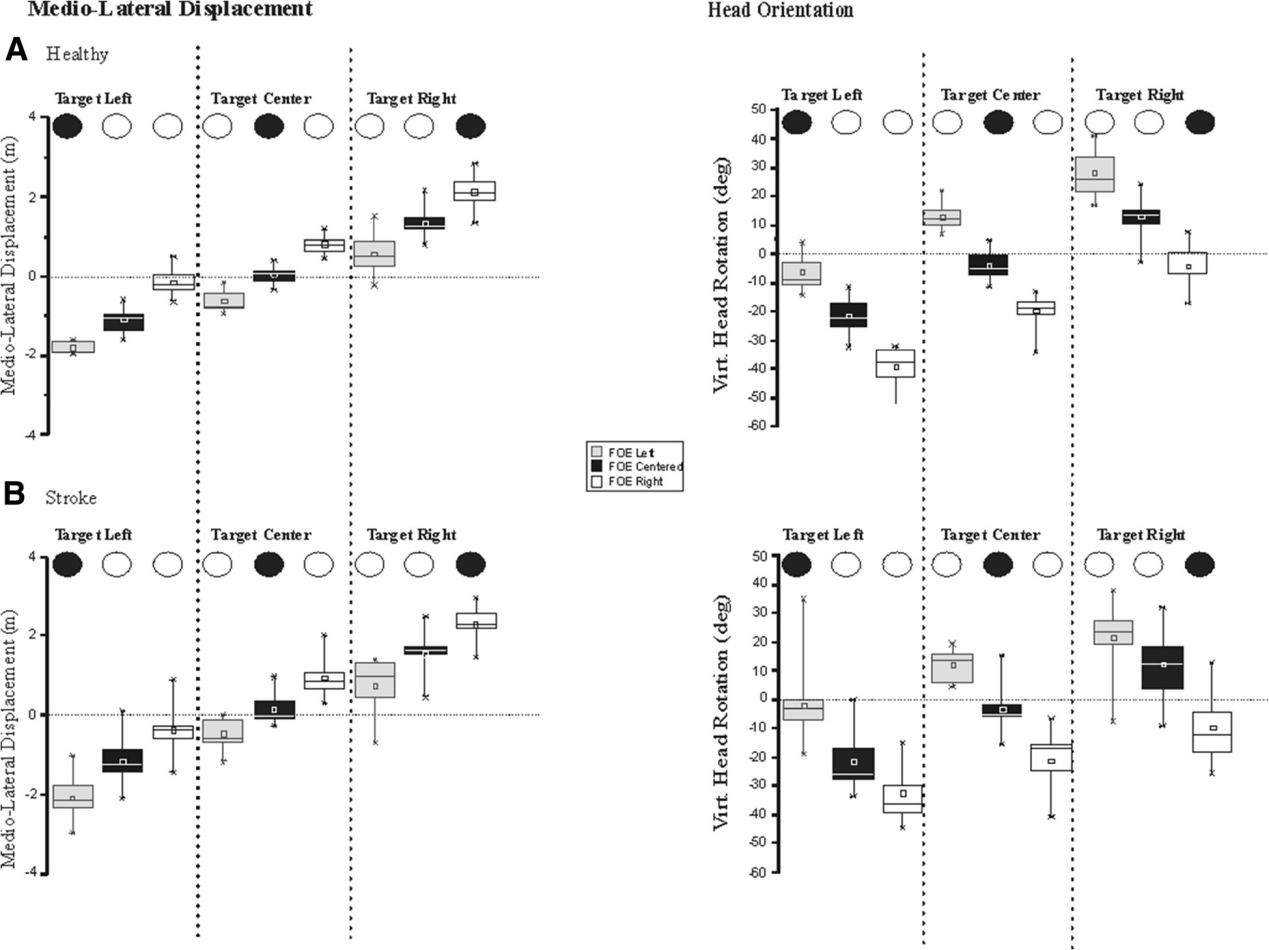
**This figure shows box and whisker plots of medio-lateral displacements (left panel) and head reorientation (right panel) across target and FOE locations for the healthy (A) and stroke (B) participants.** The body of the boxes represents the first and third quartile while the middle horizontal line and open squares within the boxes represent, respectively, the median and mean of the data set. The whiskers extend from the lowest to the highest values of the data set.

Figure 3 shows CoM ML displacement and head orientation in the virtual world coordinates for all stroke and healthy control participants. In both groups, a clear effect of target and FOE location on the extent of ML displacement and head reorientation was observed. First, the virtual ML displacement and, in most instances, virtual head reorientation, occurred in the direction of the target shift. The direction of the FOE shifts, however, modulated the extent of these adjustments. For example, when the target and FOE were shifted in the same direction (ipsilateral shifts), participants were being ‘pulled’ toward the FOE and ended up facing the target located 2 m to the side. This led to large virtual ML displacements towards the direction of the target, with little head reorientation needed to be aligned perfectly with the target. In contrast, when the FOE was shifted contralaterally to the target shift, participants were ‘pulled’ away from the target, leading to small virtual ML adjustments that were compensated by large head reorientations. When the flow remained centered, targets shifts resulted in ML deviations and head reorientations towards the target, which amplitudes were in between that of the ispsi- and contralateral conditions.

Overall, body ML displacements were significantly affected by FOE and target locations (FOE X target interaction, P = 0.000). Post hoc analyses revealed that there was a significant difference in ML displacement when the flow and/or target were shifted right or left compared to when it remained centered (P < 0.005), with the largest difference being observed when the flow shifted toward the left and the target was on the right or the left (P < 0.005). Although assumptions for parametric statistical analyses were not met, it can be observed that head reorientation was also affected by FOE and target. Stroke participants behaved similarly to the healthy group in terms of their ML displacement and head reorientation responses to the FOE and target shifts (no Group effect, P > 0.05). However, amplitudes of corrections varied across the participants, resulting in larger variability in the stroke group compared to the healthy controls.

### Net heading errors

**Figure 4:**
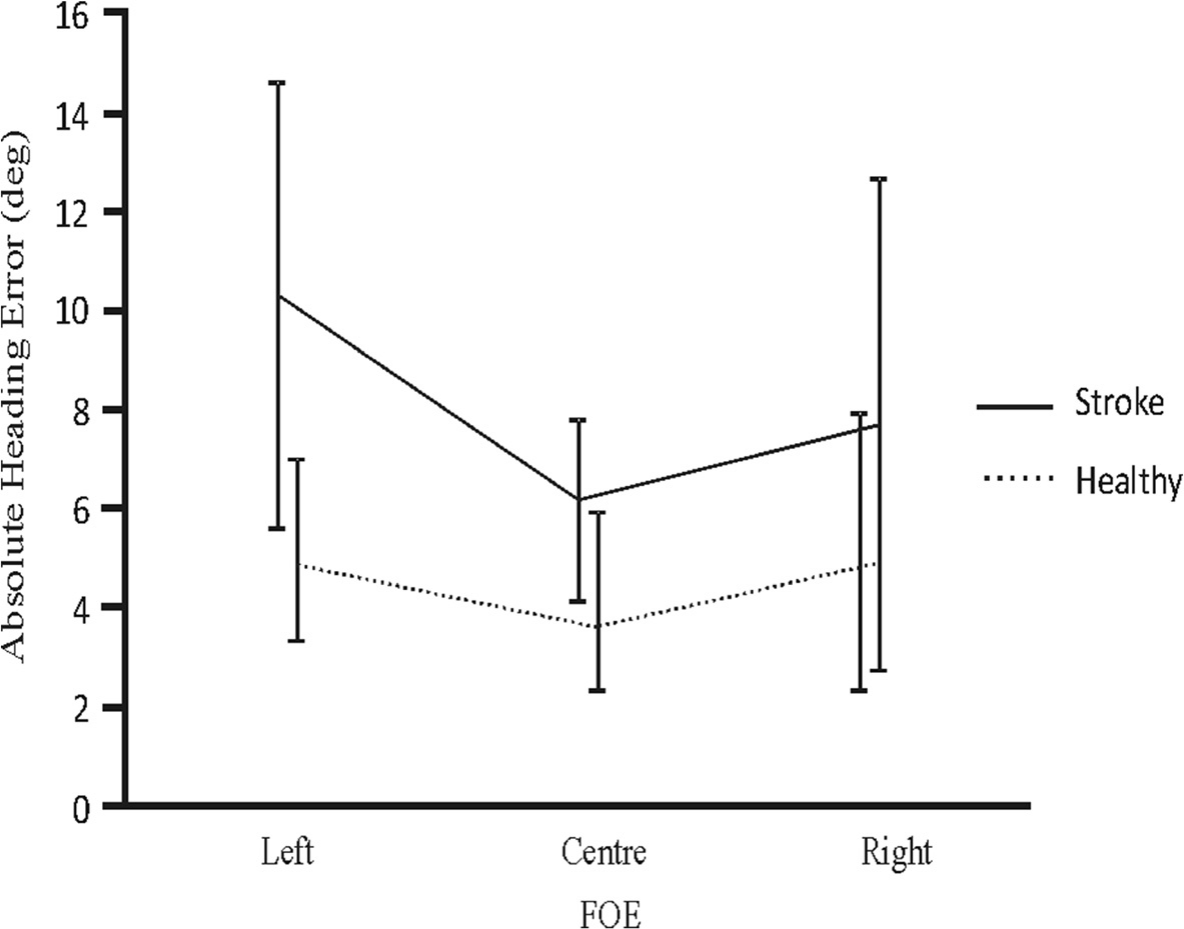
**Mean (±1SD) absolute heading errors across focus of expansion (FOE) locations in stroke participants and healthy controls.** Since no significant effects of target location were observed, data for the different target locations were pooled altogether.

NHEs at 5 m of forward walking were calculated, based on CoM ML displacement and head orientation (see Methods). Results indicate that participants generally overshot and undershot when the FOE and the target were located ipsilaterally and contralaterally, respectively. NHEs were within 8.11° and 2.91° for the stroke participants and healthy participants, respectively. Since some of the participants undershot whereas others overshot under the same conditions, absolute NHEs that ignored the direction of the error were analyzed and compared between groups and across conditions. Statistically significant effects for ‘Group’ (P = 0.011) and ‘FOE’ (P = 0.001) were observed, with a significant interaction between Group and FOE (P = 0.04) (Figure 4). Target location had no impact on absolute NHEs. Post hoc comparisons revealed that there were no significant differences between groups when the FOE was shifted to the right/paretic side or remained in the center (P > 0.05). However, stroke participants, but not healthy controls, made larger errors when the flow shifted to the left/non-paretic (P < 0.05). This was especially pronounced in patients S2 and S3 who sustained a right CVA (see Table 1).

### Heading and head reorientation onset

No differences were observed between the stroke and healthy participants (Group, P > 0.05) for the onset of head and heading reorientation. Head reorientation preceded heading reorientation when the target was shifted to the side, regardless of FOE location, both for healthy (head = 0.71 ± 0.14 s, heading = 0.83 ± 0.49 s) and stroke participants (head = 0.98 ± 0.09 s, heading = 2.05 ± 0.78 s). However, when the target remained centered but the FOE was shifted, which caused the participants to make adjustments in response to the flow, heading reorientation preceded head reorientation in the healthy (0.53 ± 0.00 s vs. 1.32 ± 0.16 s) as well as the stroke participants (1.35 ± 0.19 s vs. 2.87 ± 0.01 s). There was an overall main effect due to the FOE location on the onset of head and heading reorientation (P < 0.05). Reorientation of heading and head occurred later when the FOE was shifted right or left compared to the FOE centered position.

### Gait speed and clinical outcomes

Although healthy control participants were assessed at slow walking speed in an attempt to match the speed of the stroke participants, there was a significant difference in gait speed between the two groups (t test, P < 0.05) during the walking task. Pearson Correlation Coefficients revealed no significant relationship between the absolute NHEs and gait speed during the actual walking task or as measured by the 10 m walk test in both groups. Similarly, no relationships were found between absolute NHEs and performance on the Trail Making B.

### Seated steering experiment

**Figure 5:**
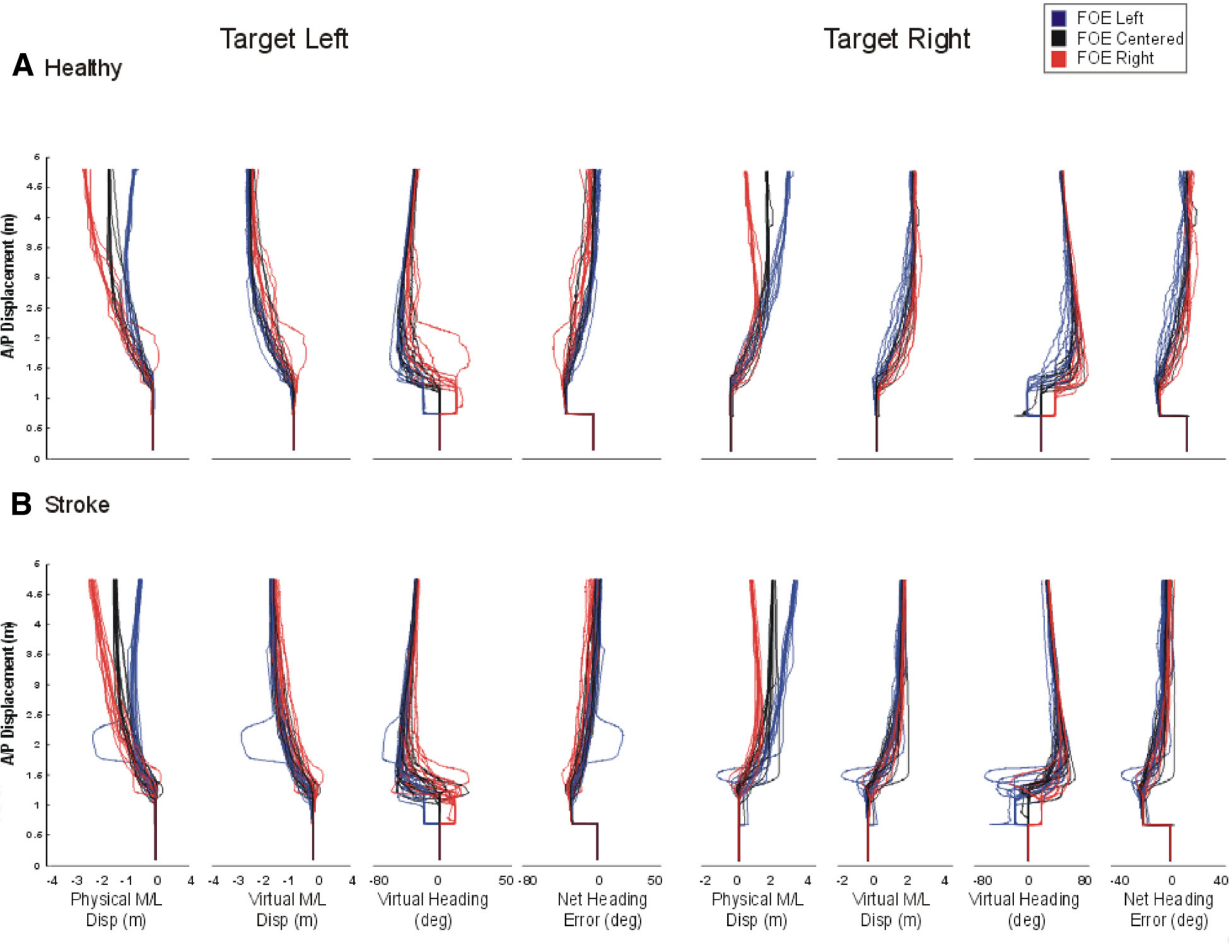
**Steering behaviors for one healthy and one stroke participant (S5) exposed to target shifts towards the left and right under optic flows of different focus of expansion (FOE) locations while performing a steering task with a mouse while seated.** A/p: anterio-posterior, M/L: Mediolateral, Disp: Displacement, Deg: Degree, FOE: focus of expansion.

**Figure 6:**
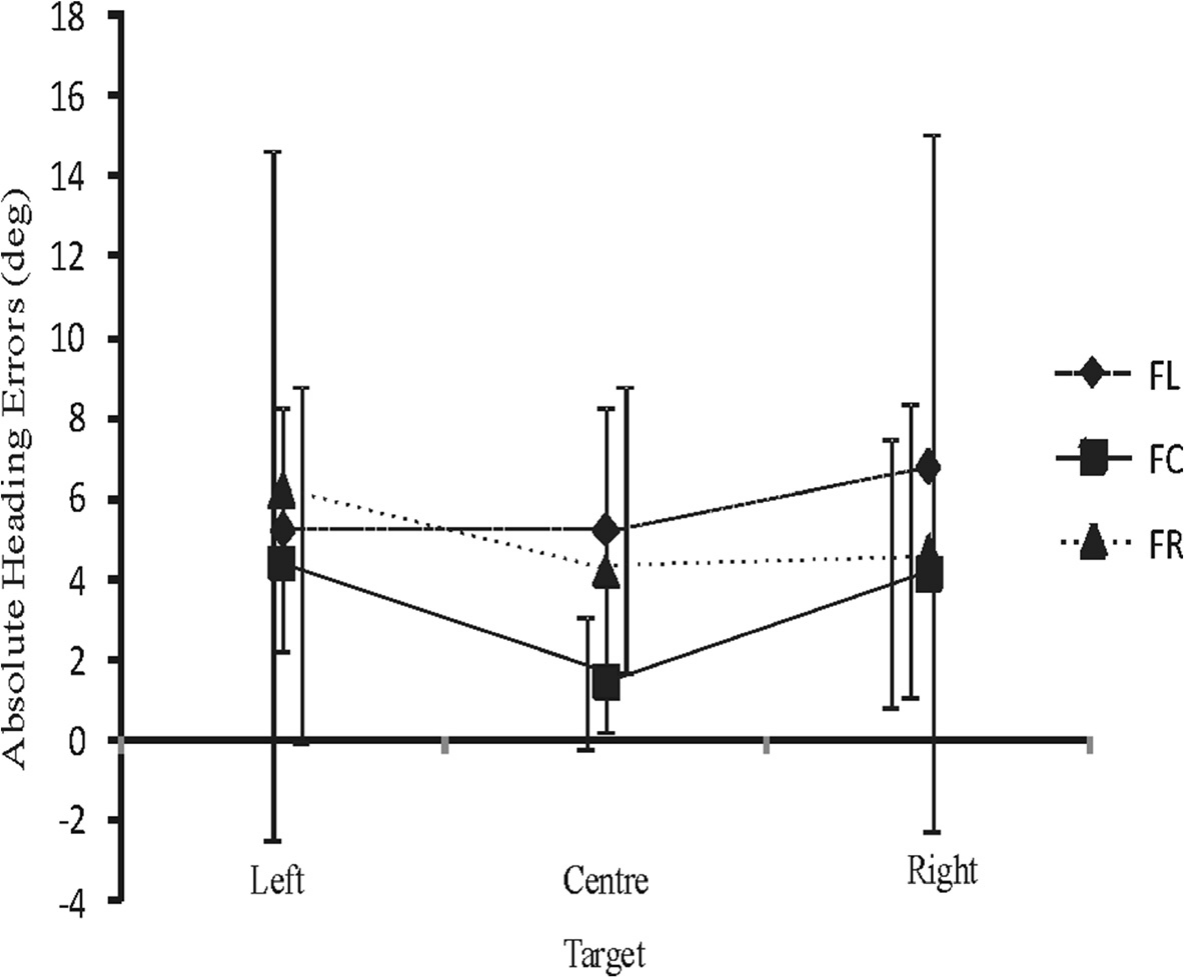
Mean (± 1SD) absolute heading errors across focus of expansion (FOE) locations and target locations in stroke and healthy participants together.

Individual examples of behaviors for the steering task performed while seated are presented for one healthy and one stroke participant exposed to target shifts towards the left and right with different FOE locations (Figure 5). As for the walking experiment, a target shift towards the left, for instance, induced a change of trajectory towards the left, both in the physical and the virtual world coordinates. The direction of the flow modulated the extent of the leftward deviation, with contralaterally vs. ipsilaterally located FOEs with respect to the target resulting in larger vs. smaller leftward physical ML displacements, respectively. The magnitude of ML correction in the physical world was such that corresponding trajectories and heading in the virtual world coordinates were very similar and resulted in small NHEs across conditions. A repeated measure ANOVA revealed that there was no significant difference in absolute NHEs between stroke (range: 1.16°-9.07°) and healthy (range: 1.8°-5.28°) participants (P = 0.436). A main effect of FOE (P = 0.001) and target (P = 0.007) on absolute NHEs in the seated steering task was also observed, with larger errors being observed with shifted FOEs and shifted targets compared to the centered conditions (Figure 6). For this seated steering task where the participant’s perceived position in the VE was controlled with ML mouse movements, all differences in NHEs can be attributed directly to differences in ML displacement. There was no correlation between NHEs in sitting and walking for any of the tested conditions (P > 0.05).

### Heading reorientation onset

No differences in heading reorientation onset were observed between the two groups of participants and due to changes in target location. A main effect due to the FOE location, however, was observed on the onset of heading reorientation (P < 0.000), with delayed onsets being observed with shifted FOEs (healthy: 0.19 ±0.25 s; stroke: 0.22 ± 0.22 s) compared to the FOE centered condition (healthy: 0.11 ±0.007 s; stroke 0.17 ± 0.018 s).

## Discussion

This study is the first to examine the interaction of changing optic flows and target locations on locomotor steering strategies in stroke and healthy participants. Results demonstrate that participants used steering strategies that combined both body ML displacement and head in space reorientation to align themselves with the target. Optic flow and target shifts modulated the direction and/or magnitude of ML trajectory and head orientation adjustments, as well as the timing and sequence of head and heading reorientation. Compared to healthy controls, stroke participants displayed larger NHEs in the walking steering task but not in the seated steering task, suggesting that an altered perception or visuomotor transformation of visual motion/target location is not a main contributing factor.

### Changes in optic flow direction modulate steering strategies

As anticipated, present findings show that steering towards shifted targets is accomplished using both trajectory changes (translational component) and changes in head/body in space orientation (rotational component) in the direction of the goal, as demonstrated in earlier reports [17,18]. Our results also revealed, however, a strong modulation effect of optic flow direction on the weighting of these translational and rotational components, as well as on their sequencing. Indeed, while ipsilateral FOE shifts (to the target shifts) lead to large changes in ML trajectory with small head reorientations, contralateral FOE shifts rather lead to small ML adjustments accompanied by large head reorientations. Such modulation pattern can be entirely explained in biomechanical terms. As ipsilateral FOE shifts ‘pulled’ the participants in the same direction as the target shift in the virtual world, a large virtual ML displacement arose, leading the participants to face the virtual target. Little head orientation adjustments were therefore needed for the participants to be aligned with the target. With contralateral FOE shifts, the participants who were being pulled in the direction opposite to the target shift counteracted this deviation by translating in the opposite direction in the physical world (see displacement traces in the physical world). This translation, however, barely surpassed the opposite effect of the FOE shift and resulted in a small net displacement in the virtual world. As a result, a large head reorientation in the direction of the target was executed. While this leads to an overshoot and undershoot of net heading corrections for ipsilateral and contralateral FOE/target shifts, the net heading errorsremained of small magnitude, at least in the healthy group. Altogether, these findings emphasize the interdependence and fine coordination between rotational and translational steering mechanisms while walking.

The present study, which was the first to examine the effects of flow shifts on the onsets of kinematic changes while steering towards a target, also revealed the surprising finding of a change in the sequence of head and heading reorientation. Changing direction while walking is normally accomplished by a horizontal reorientation of eye/head in the direction of the future travel path, which precedes the change in heading or body trajectory [17-19]. In the present study, a target shift caused the participants to rotate their head and then readjust their heading, which is consistent with earlier observations. When the target remained centered and the FOE was shifted to the side, however, heading reorientation preceded head reorientation. A tentative explanation is that the presence of a target shift, in addition to draw the participant’s attention, also allows for a gaze reorientation, which was suggested to be a pre-requisite to elicit an anticipatory steering synergy [20]. At variance, a gaze fixation on the central target during the target centered condition, regardless of the FOE location, might have prevented this synergy to appear, resulting in delayed head reorientation.

### Stroke alters steering strategies

Results in the stroke participants are characterized by the presence of larger net heading errors. As in their healthy counterparts, head and body ML displacement were consistently directed towards the target, despite the presence of an optic flow shift. A previous study from our laboratory has shown that stroke participants veered in the wrong direction in response to the FOE shifts [12]. In the present study, however, participants responded by veering toward the desired direction, a difference that may be attributed to the presence of a target. Such target might have provided a source of visual information of lesser complexity compared to higher-order visual motion information. The smaller magnitude of the steering adjustments of the stroke participants led to larger heading errors, a finding that can be attributed to a lack of head reorientation rather than to smaller body ML displacements.

Results also revealed that stroke participants displayed especially large net heading errors when the flow was shifted to the left/non-paretic side, an observation that was even more pronounced for participants S2 and S3. Such failure to adequately respond to non-paretic FOE shifts has been observed previously and was proposed to be the results of persistent visuospatial neglect and/or far extra-personal space neglect that might not have been picked up by paper and pencil tests [12,13]. In favor of this hypothesis, both participants S2 and S3, who were the only participants with a right CVA, did present with a history of neglect that was apparently completely resolved based on the paper and pencil screening tests used in this project. Altogether, these results seem to support previous findings that people with neglect have altered steering strategies [21-23].

While the fact that the two patients with a history of neglect displayed the most altered performance points towards the contribution of a spatial-attentional disorders, it is interesting to note that the absolute net heading errors were affected in the walking task, but not in the seated steering task. It could be argued that an interaction between handedness and side of the CVA may have influenced this finding, as most stroke participants performed the seated steering task with their non-dominant hand. Such possibility cannot be fully excluded, despite the full block of practice trials provided before data collection and the increased use of the non-paretic arm normally experienced after stroke. If anything, one can assume that stroke participants would have performed even better, had they executed the task with the dominant hand (which was not an option), hence further minimizing differences between the two experimental groups.

Locomotion is a complex task which requires a high level of sensorimotor integration and entails the completion of several sub-tasks, including the maintenance of the body against gravity, balance and the control of speed and direction of progression, all of which can be affected by stroke. It can be assumed that these challenges were minimized in the seated steering task, as the person was seated and performed the task with the non-paretic arm. The walking task also presented more degrees of freedom than the seated steering task, with the latter allowing only for ML displacement, as compared to both displacement and head/body reorientation in the walking task. On the one hand, the preserved performance of the stroke participants in the seated steering task suggests that an altered perceptuo-motor processing of optic flow information is not the most likely candidate to explain their poor steering performance during walking and that other sensorimotor deficits might have contributed. Such observation is consistent with the study of Billino and collaborators, where a preserved perception of heading from optic flow was observed in 22 out of 23 stroke participants who presented with a variety of brain lesions [15]. On the other hand, given the complexity of locomotion, it cannot be excluded that the damaged central nervous system might have prioritized the completion of sub-tasks of gait judged as essential (e.g. maintenance of body against gravity, balance and progression) at the expense of interpreting complex visuo-spatial information.

### Limitations

The first limitation of this study is the absence of a purely perceptual task, as opposed to the perceptuo-motor task (seated steering task) examined in this study. Such a perceptual task could have provided even stronger evidence of a potentially preserved perception of visual motion and target location in the stroke participants. Another limitation is the difference in gait speed between the stroke and healthy participants, despite of having healthy controls walk at slow speed. No relationships, however, were found between the magnitude of NHEs and gait speed. In addition, slower speeds of optic flow were shown to yield lower discrimination thresholds (better performance), possibly because it facilitates the use of position information [24]. These speed-induced effects, however, were observed for large speed increments and a range of speed values (5 m/s, 15 m/s and 26 m/s) exceeding by far that observed in this study (<1 m/s). Furthermore, the participants’ discrimination performance approached perfection (90-100% correct) at 5 m/s, such that one can question whether further improvements would be observed at slower speeds. Based on these observations, it is not likely that a meaningful change in performance can be attributed to speed differences between the groups (or even tasks). In the unlikely event of an effect induced by the small speed differences observed in this study, it would favour a better performance during walking compared to the seated task, and a better performance in the stroke participants compared to the healthy controls. Finally, the limited sample size and predominance of left CVA within the group of stroke participants examined in this study may limit the generalization of findings to the a stroke population that present with different characteristics (e.g. lesion side, level of functional capacity, etc.).

## Conclusion

Participants used a hybrid strategy, which combined both translational and rotational adjustments of their head/body to align themselves with targets shifted to the side. A strong modulation effect of optic flow direction on the weighting of these translational and rotational components, as well as on their sequencing, was observed. The larger heading errors in stroke participants for the steering task in walking but not while seated suggests that an altered perception of visual information is not a main factor to their poor locomotor steering abilities. It cannot not be excluded, however, that subtle changes in the processing of visual information takes place while walking, as resources during such a complex task may be directed towards accomplishing other priority sub-tasks, such as the maintenance of upright posture, balance and forward progression.

## Abbreviations

HMD: Helmet mounted display; VE: Virtual environment; OP: Optic flow; FOE: Focus of expansion; ML: Mediolateral; CoM: Center of mass; NHE: Net heading error.

## Competing interests

The authors declare that they have no competing interests.

## Authors’ contributions

AA and AL conceived and carried out the experiment and analyses presented in this manuscript. Both authors contributed to the manuscript preparation and read and approve the final manuscript.
